# ACSL1-induced ferroptosis and platinum resistance in ovarian cancer by increasing FSP1 N-myristylation and stability

**DOI:** 10.1038/s41420-023-01385-2

**Published:** 2023-03-08

**Authors:** Qingyu Zhang, Ning Li, Limei Deng, Xingmei Jiang, Yuming Zhang, Leo Tsz On Lee, Haitao Zhang

**Affiliations:** 1 Laboratory of Obstetrics and Gynecology, Affiliated Hospital of Guangdong Medical University, Zhanjiang, Guangdong, 524001 China; 2 The Marine Biomedical Research Institute, Guangdong Medical University, Zhanjiang, 524023 China; 3 Department of Hematology, Affiliated Hospital of Guangdong Medical University, Zhanjiang, Guangdong, 524001 China; 4 Cancer Centre, Faculty of Health Sciences, University of Macau, Taipa, Macau, China; 5 Ministry of Education Frontiers Science Center for Precision Oncology, University of Macau, Macau, China; 6 Peptide and Protein Research and Application Key Laboratory of Guangdong Medical University, Zhanjiang, Guangdong, 524023 China; 7 Department of Biochemistry and Molecular Biology, Guangdong Medical University, Zhanjiang, Guangdong, 524023 China

**Keywords:** Gynaecological cancer, Cancer metabolism

## Abstract

Reprogramming of lipid metabolism, which modulates energy utilization and cell signaling, maintains cell survival and promotes cancer metastasis in cancer cells. Ferroptosis is a type of cell necrosis caused by an overload of lipid oxidation, which has been demonstrated to be involved in cancer cell metastasis. However, the mechanism by which fatty acid metabolism regulates the anti-ferroptosis signaling pathways is not fully understood. The formation of ovarian cancer spheroids helps to counteract the hostile microenvironment of the peritoneal cavity with low oxygen, shortage of nutrients, and subjected to platinum therapy. Previously, we demonstrated that Acyl-CoA synthetase long-chain family member 1 (ACSL1) promotes cell survival and peritoneal metastases in ovarian cancer, but the mechanism is still not well elucidated. In this study, we demonstrate that the formation of spheroids and under exposure to platinum chemotherapy increased the levels of anti-ferroptosis proteins as well as ACSL1. Inhibition of ferroptosis can enhance spheroid formation and vice versa. Genetic manipulation of ACSL1 expression showed that ACSL1 reduced the level of lipid oxidation and increased the resistance to cell ferroptosis. Mechanistically, ACSL1 increased the N-myristoylation of ferroptosis suppressor 1 (FSP1), resulting in the inhibition of its degradation and translocation to the cell membrane. The increase in myristoylated FSP1 functionally counteracted oxidative stress-induced cell ferroptosis. Clinical data also suggested that ACSL1 protein was positively correlated with FSP1 and negatively correlated with the ferroptosis markers 4-HNE and PTGS2. In conclusion, this study demonstrated that ACSL1 enhances antioxidant capacity and increases ferroptosis resistance by modulating the myristoylation of FSP1.

## Introduction

Lack of nutrient supply to the inner core of cancer spheroids induces cancer cells to undergo reprogramming of lipid metabolism to meet energy needs and alter cell signaling favoring cell survival [1]. Massive amounts of reactive oxygen species (ROS) are generated during cancer spheroid formation and platinum-based chemotherapy [2, 3]. Cancer cells that have iron-addictive characteristics accelerate the “Fenton reaction” and generate an overwhelming accumulation of lipid peroxides that contribute to ferroptosis [4]. Furthermore, platinum-based drugs also increase ROS levels by efficiently reducing glutathione (GSH) levels and altering the redox balance of cells during chemotherapy [1, 5]. However, cancer cells can escape these ROS stresses by activating an antioxidant pathway and, therefore, acquiring resistance to platinum.

Ferroptosis is a regulated cell death mechanism (RCD) that is caused by the iron-dependent accumulation of lipid peroxides [6]. Dysfunction of an antioxidant system, such as the cystine-glutamate antiporter/Glutathione/Glutathione Peroxidase 4 (Xc-/GSH/GPX4) axis, could also result in ferroptosis. SLC7A11/GPX4 is a classic signaling pathway activated in the defense of cellular antioxidation [7]. Furthermore, recent reports have suggested that activation of Apoptosis-Inducing Factor Mitochondrion-Associated 2(AIFM2), a GPX4 independent enzyme, plays a critical role in peroxide lipid detoxification, contributes to ferroptosis resistance, and therefore has been renamed ferroptosis suppressor 1 (FSP1) [8]. Cell ferroptosis sensitivity is closely related to lipid composition. Cells with a level of polyunsaturated fatty acids (PUFA) in phospholipids, for example arachidonic acid, react with ROS and initiate ferroptosis [9]. On the contrary, upregulation of stearoyl-CoA desaturase 1 (SCD1), which increases the production of monounsaturated fatty acids (MUFA) phospholipids, could reduce ROS-induced stress. Cancer cells could also be resistant to ferroptosis by increasing the synthesis of unsaturated plasmalogens in the peroxisome [10]. However, it is not yet clear whether metabolic reprograming of cellular lipids may directly regulate the intracellular antioxidant system and therefore affect ferroptosis sensitivity.

Members of the long-chain family Acyl-CoA synthase (ACSL) are key enzymes in the regulation of lipid metabolism, including fatty acid elongation, oxidative decomposition, phospholipid generation, and protein acylation [11]. ACSL3 mediates phospholipid biosynthesis using exogenous monounsaturated fatty acids and inhibits cell ferroptosis [12]. ACSL1, like ACSL3, may inhibit ferroptosis by mediating the integration of exogenous linolenic acid into cell phospholipids [13]. Our recent study suggested that ACSL1 and ACSL3 protein levels are significantly up-regulated in the highly metastatic ovarian cancer cell lines and their gene expressions are correlated with metastatic capacity and poor survival prognosis [14]. ACSL1 overexpression significantly improves proliferation of ovarian cancer cells, induces cancer spheroid formation, and tumorigenesis in xenograft model through enhanced myristic acid (MA) production and therefore increased protein N-myristoylation.

In this report, we reveal that ACSL1 induction during spheroid formation and platinum-based chemotherapy could suppress ROS and ferroptosis by increasing myristoylation of FSP1 and developing ferroptosis and drug resistance. Therefore, our study improves the current understanding of the underlying mechanisms responsible for lipid metabolism reprogramming to enhance ferroptosis resistance in ovarian cancer and sheds light on the prevention of ferroptosis resistance that develops during platinum-based chemotherapy.

## Results

### Ferroptosis resistance contributed to ovarian cancer spheroid formation

**Fig. 1:**
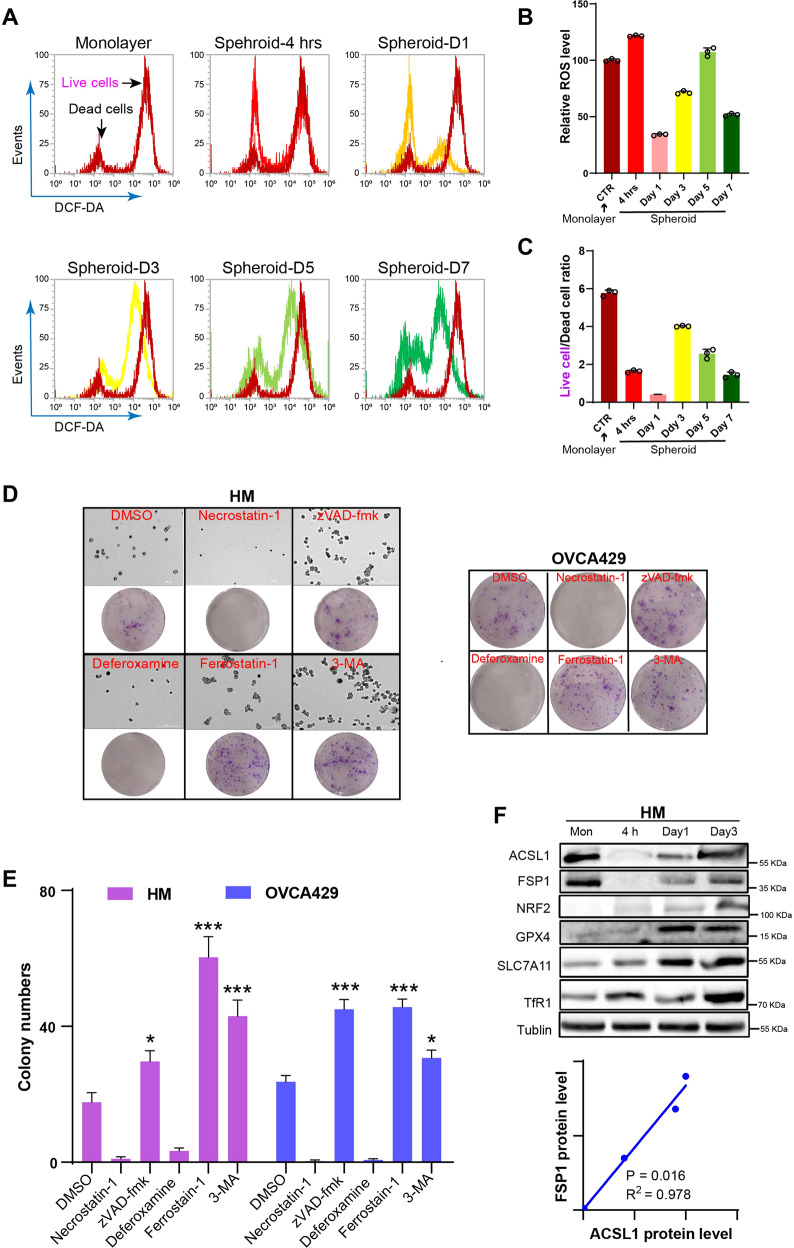
Ferroptosis is involved in the formation of spheroids in ovarian cancer. **A** The ROS level of cell monoculture and different spheroid formation events were labeled by DCFHDA and detected by flow cytometry. Dynamic changes in ROS signal intensity between monoculture cells and different time points for spheroid formation. **B** Statistical analysis of ROS levels of live cells in monoculture at different time points of spheroid formation. **C** The ratio of live cells to dead cells in each group was calculated according to the amount of ROS in Fig. 1B. **D** The morphology of the formation of the spheroid after the addition of various inhibitors and colony formation was observed under 10x objective lens and live spheroid was evaluated after reseeding the spheroid of HM cells and OVCA429 cells in normal 6-well culture plates for 7–10 growth and stained with crystal violet. **E** Statistical analysis of the number of spheroids after the addition of various inhibitors to HM cells and OVCA429 cells. **F** The expression of ferroptosis-related proteins and ACSL1 in monoculture cells and spheroids of different time was detected by western blotting. *P < 0.05 and ***P < 0.001 indicate statistical significance.

Ovarian cancer cells undergo “anoikis” after losing their epithelial state during peritoneal metastasis, which leads to oxidative stress [16, 17]. We used DCFHDA labeling to study the dynamic changes of ROS levels at different time points during the formation of the spheroids. ROS levels of suspended cells temporarily increased at 4 h and resulted in a significantly reduced number of live cells due to anoikis (Fig. 1A, B). Interestingly, during spheroid formation (after day 1), ROS levels gradually increased from day 1 to day 3 due to hypoxia and limited energy supply during spheroid growth; however, on day 7, the ROS levels decreased dramatically. The number of surviving cells had not expanded, and ROS levels remained as low as on day 1, the ROS levels at 4 h and on day 5 were higher but the live cell ratio was lower (Fig. 1C) These data indicated that high levels of ROS were harmful to cancer cell survival and overcoming ROS accumulation enhances the formation of cancer spheroids.

To explore cell death mechanisms, inhibitors of apoptosis (ZVAD-FMK), necroptosis (necrostatin-1), autophagy (3-MA), and ferroptosis (ferrostain-1) were applied during spheroid formation of HM and OVCA429 cells. Inhibition of apoptosis, autophagy, and ferroptosis significantly enhanced spheroid formation, while the necroptosis inhibitor did not enhance spheroid formation (Fig. 1D, E). This suggested that ovarian cancer spheroid growth is limited by cell apoptosis, autophagy, and ferroptosis. The iron chelator deferoxamine mesylate did not inhibit ferroptosis-enhancing spheroid formation, but inhibited spheroid formation. However, iron is indispensable, which is in line with a previous report in which ovarian cancer was reported to require iron to support ovarian cancer stem cell maintenance and cell growth, a phenomenon called “iron addiction” [4].

To verify the role of the antioxidant pathway in the balance of ROS accumulation and ferroptosis during spheroid formation, the expression of antioxidant pathway-related proteins, including nuclear factor erythroid 2-related factor 2 (NRF2), glutathione peroxidase 4 (GPX4), and SLC7A11 solute carrier family 7 member 11 (SLC7A11), as well as proteins in the regulation of ferroptosis, FSP1 and transferrin receptor 1 (TfR1), were verified by western blotting (Fig. 1F). All proteins tested were upregulated during spheroid formation. The data suggested that activation of this antioxidant pathways could be a key factor in maintaining the survival of spheroid cells.

### Up-regulation of ACSL1 reduced lipid peroxidation and promoted ovarian cancer spheroid formation through lipid reprogramming

**Fig. 2:**
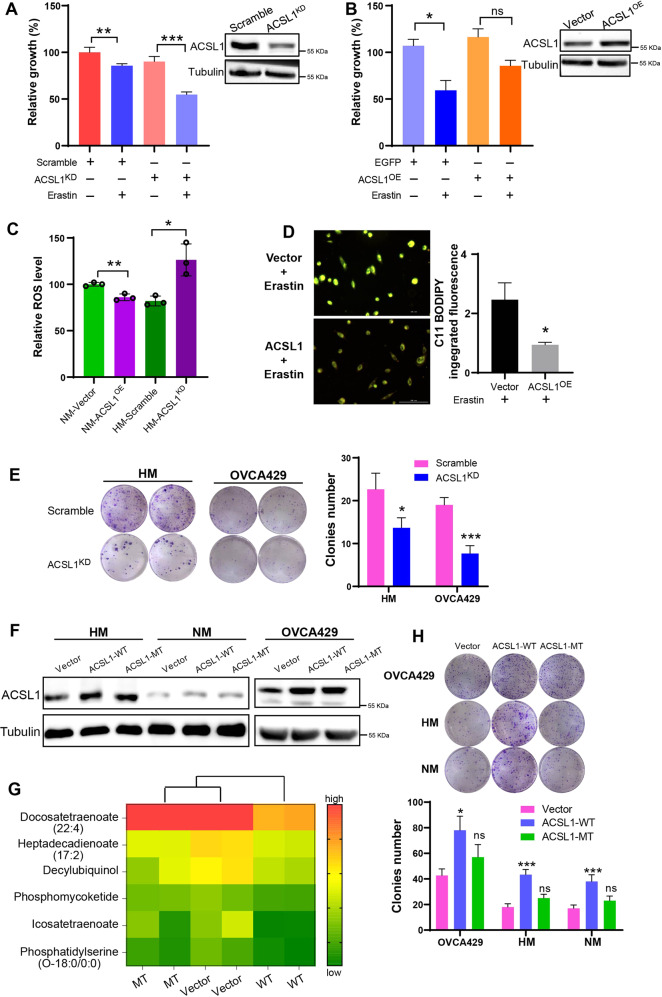
ACSL1 upregulates SLC7A11 and FSP1 inhibits ferroptosis. **A**, **B** The CCK8 assay was performed to detect cell viability after ACSL1 knockdown or overexpression. **C** Changes in ROS levels after ACSL1 overexpression in NM cells and ACSL1 knockdown in HM cells were detected by the DCFDA assay. **D** C11 BODIPY was used to detect peroxided lipid after ACSL1 overexpression in NM cells. The reduced state of C11 BODIPY is orange, after oxidation it switches to green; therefore, yellow represents the level of oxidation. The fluorescence was observed under 10x objective lens of Cytation 5 Cell Imaging Multimode Reader. **E** After the knockdown of ACSL1 from HM cells and OVCA429 cells, the spheroid formation capacity was assayed. **F** Wild type ACSL1 (ACSL1-WT) and mutant ACSL1 (ACSL1-MT) were overexpressed in NM, HM, and OVCA429 cells, respectively, and its expression was confirmed by western blotting. **G** Lipid metabolomics analysis of lipidome changes in NM cells after overexpression of ACSL1-WT and ACSL1-MT. The heatmap shows the differential lipid composition between the wild type and control groups. **H** Spheroid formation capacity of cells after overexpression of ACSL1-WT and ACSL1-MT in NM cells and OVCA429 cells. *P < 0.05, **P < 0.001, and ***P < 0.001 indicate statistical significance.

Our previous study suggested that ACSL1 promotes ovarian cancer metastasis, and the above data also demonstrated activation of the antioxidant pathway during spheroid formation. Hence, we proposed that ACSL1 may be responsible for the reduction of ROS and could suppress ferroptosis during spheroid formation. We established a HM cell line harboring silenced ACSL1 siRNA gene (ACSL1^KD^) expression or the transient overexpression of ACSL1 (ACSL1^OE^) in NM cells. The results suggested that the ferroptosis inducer (Erastin) only slightly decreased the cell growth of HM cells in the control group, but the elimination of ACSL1 significantly increased ferroptosis and suppressed the spheroid growth of HM cells (Fig. 2A). Meanwhile, Erastin has a stronger inhibitory effect on NM cells, and ACSL1^OE^ could partially increase the effect of Erastin (Fig. 2B). This indicated that the level of ACSL1 in cancer cells could significantly modulate ferroptosis and cell growth. When we analyzed the ROS levels, as expected, the expression of ACSL1 was correlated with intracellular ROS levels; as the ROS level decreased significantly in NM-ACSL1^OE^ cells and increased in HM-ACSL1^KD^ cells (Fig. 2C). The reduction in lipid peroxidation was confirmed by C11 BODIPY staining. A significant reduction in lipid peroxidation by erastin was found in NM-ACSL1^OE^ cells (Fig. 2D). Furthermore, the elimination of ACSL1 also suppressed the ability to form cancer spheroids in OVCA429 and HM cells (Fig. 2E).

S277 and K675 in ACSL1 have been reported to be the binding sites of ATP, which is a crucial factor for catalyzing fatty acid activation (Acyl-CoA). Binding ACSL1 S277 and K675 were thus mutated to alanine to inhibit ACSL1 activity by reducing ATP binding [18]. The wild-type proteins (ACSL1-WT) and mutant (ACSL1-MT) were stably overexpressed by lentivirus infection in NM cells, OVCA429 cells, and HM cells, respectively (Fig. 2F). Metabolic profiles were compared, and the data suggested that ACLS-WT altered the lipid profile, by especially significantly reducing docosatetraenoate levels (a PUFA that renders cells more sensitive to ferroptosis stress), when compared to ACSL-MT and vector control cells (Fig. 2G). Functionally, ACSL1-WT significantly increased spheroid formation in all cell lines tested, while ACSL1-MT overexpression did not produce any significant changes (Fig. 2H).

### ACSL1 specifically upregulated FSP1 by blocking the proteosome degradation pathway

**Fig. 3:**
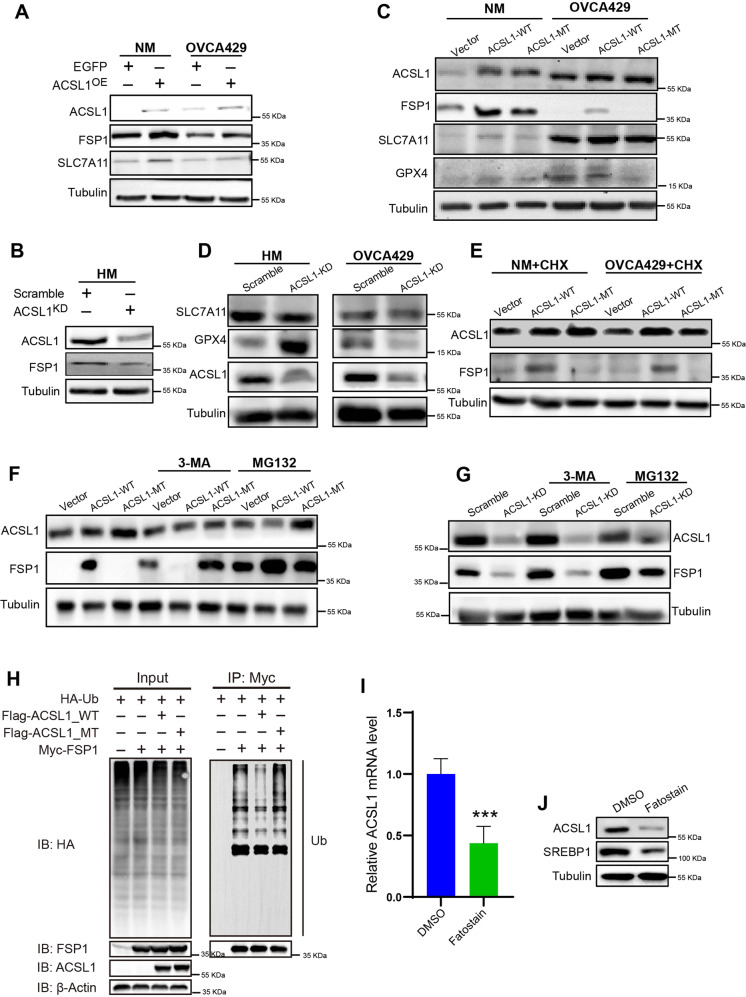
SREBP1/ACSL1/FSP signaling pathway regulation mechanism in ovarian cancer. Expression of antioxidant-related proteins SCL7A11, FSP1 after (**A**) transient overexpression of ACSL1 in NM cells and OVCA429 cells and (**B**) knockdown of ACSL1 in HM cells. **C** Overexpression of ACSL1-WT and ACSL1-MT in NM and OVCA429 cells and protein levels of SLC7A11 and GPX4. **D** After ACSL1 was knocked out in HM cells and OVCA429 cells respectively, ACSL1, SLC7A11, and GPX4 were detected by western blotting. **E** The protein level of FSP1 following CHX inhibitor treatment. **F** After overexpressing ACSL1 and its mutants in OVCA429 ovarian cancer cells, respectively, FSP1 expression was detected by western blotting of cells in the presence or absence of the autophagy blocker 3-MA and the proteasome inhibitor MG132. **G** After the knockdown of ACSL1 ovarian cancer OVCA429, FSP1 expression in each group was detected by western blotting when the cells were exposed to 3-MA and MG132 or untreated cells. **H** FSP1 ubiquitination in ACSL1 wild type or ACSL1 mutant overexpression in HEK293T cells was detected by immunoprecipitation. **I**, **J** Gene expression and protein levels of the SREBP1 and ACSL1 in HM cells treated with the SREBP1 small molecule inhibitor fatostatin. *P < 0.05 and ***P < 0.001 indicate statistical significance.

We then investigated the association between antioxidant-related proteins with ACSL1 in various ovarian cancer cell lines. ACSL1 overexpression in NM and OVCA429 cells not only increased the level of FSP1, but also increased the level of SLC7A11 (Fig. 3A), which mediated cystine uptake to regulate redox homeostasis [19]. The increase in SLC7A11 indicated a balance of higher ROS levels during spheroid formation. Meanwhile, the silencing of ACSL1 in HM cells could reduce the protein levels of FSP1 and SLC7A11 (Fig. 3B), which also suggested the role of ACSL1 in upregulating the antioxidant pathway. The effects of ACSL1 were further confirmed by the ACSL1 mutation. FSP1 levels were increased in ACSL1-WT cells rather than ACSL1-MT (Fig. 3C). In alignment with the overexpression data, the levels of FSP1 and SLC7A11 decreased in ACSL1^KD^ cells (Fig. 3B, D). GPX4 levels were partially up-regulated by ACSL1 overexpression in NM cells, but not in OVCA429 cells. However, ACSL1 knockdown only reduced the level of GPX4 in OVCA429 cells, but not in HM cells (Fig. 3D). This implied that ACSL1 mainly induce anti-ferroptosis effects through the FSP1/CoQ10 pathway.

To address how ACSL1 regulated the level of FSP1, the protein synthesis inhibitor cycloheximide was used for 24 h (CHX, 20 µM). The results showed that the expression of FSP1 in the ACSL1-WT overexpression group still increased after CHX treatment in NM cells and OVCA429 cells, while the expression of FSP1 in the mutant group also decreased significantly regardless of CHX treatment (Fig. 3E). The above data suggested that ACSL1 did not regulate the synthesis of FSP1 proteins. Therefore, we proposed that ACSL1 upregulated the level of FSP1 protein by suppressing protein degradation. Since the macroautophagy-lysosome and ubiquitin-proteasome system (UPS) proteolysis pathways are the two main pathways of protein degradation [20], we therefore used an autophagy inhibitor (3-MA) and proteasome inhibitor (MG-132) to verify the role of ACSL1 in the degradation of FSP1. Compared to no treatment control, ACSL1-WT overexpression strongly increased the level of FSP1 protein, but not ACSL1-MT overexpression. After 3-MA treatment, the level of the FSP1 protein increased significantly in the vector control and ACSL1-MT; however, a dramatic decrease in the level of FSP1 was found following the overexpression of ACSL1-WT (Fig. 3F). Autophagy is known to enhance ferroptosis by reducing ferritin and releasing iron levels [21]. Our data indicated that basal FSP1 levels were suppressed by autophagy but activation of ACSL1 would not increase FSP1 expression when autophagy-triggered ferroptosis was blocked. To inhibit proteasome activity, MG132 (2 μM) was used to restore the level of FSP1 in the Vector and ACSL-MT groups in the tested cell lines. ACSL1-WT overexpression was not affected by MG132 treatment (Fig. 3G). As suggested above, the expression of FSP1 decreased with ACSL1 knockdown. This decrease in FSP1 expression was not restored after 3-MA treatment but was significantly restored after MG-132 treatment (Fig. 3G). To further determine whether FSP1 ubiquitination could be suppressed by ACSL1, we co-transfected FSP1-myc with ACSL1 and pulled-down FSP1 expression to detect its ubiquitination level. The results showed that ACSL1-WT could enhance FSP1 ubiquitination, but ACSL1-MT could not increase FSP1 ubiquitination (Fig. 3H).

ACSL1 is involved in metabolic reprogramming and helps maintain cell survival during the formation of ovarian cancer spheroids. SREBP1 is the master transcription factor in lipid metabolism, and up-regulation of SREBP1 is reported to be overexpressed in metastatic ovarian cancer [22, 23]. To determine whether ACSL1 expression is induced by SREBP1, fatostatin, a SRBEP1 inhibitor, was used to suppress the transcriptional and protein level of ACSL1 during spheroid formation of HM cells (Fig. 3I, J).

### ACSL1 enhanced the myristoylation of FSP1 and ferroptosis resistance

**Fig. 4:**
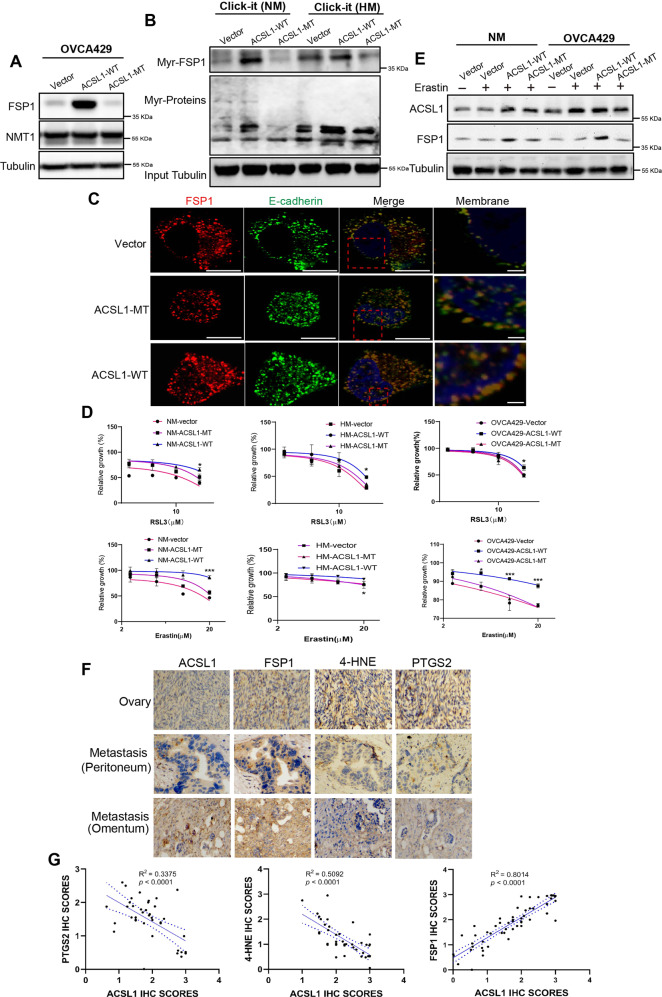
ACSL1 improves the level of myristoylation of FSP1 and increases resistance to ferroptosis in the ovarian cancer cell. **A** In OVCA429, after overexpressing ACSL1 and its mutants in the ovary, the level of NMT1 protein expression was detected by western blotting. **B** ACSL1 and its mutants were overexpressed in NM and HM ovarian cancer cells, respectively. Then myristic-azide was added to label myristoylated proteins in cells of each group and Click-it chemistry and western blotting were used to detect the expression levels of myristoylated FSP1, β-actin, and total myristoylated proteins in cells of each group. **C** Changes in protein expression of ACSL1 and FSP1 in NM and OVCA429 cells after the expression of ACSL1-WT and ACSL1-MT vectors and treatment with erastin as detected by western blotting. **D** After overexpressing ACSL1 and its mutants in OVCA429 cells of ovarian cancer, respectively, the FSP1 and E-cadherin proteins were detected in cells by immunofluorescence distribution. Scale bars indicated 10 µm and 2 µm in the overview and magnified images, respectively. **E** NM cells, HM cells and OVCA429 After wild type ACSL1 and mutant ACSL1 were overexpressed in cells, each group cell sensitive to erastin and RSL3 were detected by the CCK8 assay. **F** Immunohistochemical detection of the expression of ACSL1, FSP1, 4-HNE, and PTGS2 in normal ovarian tissue and ovarian cancer tissue samples from patients. The tissue samples were captured under a microscope at a magnification of 20x. Ovary-O represents normal ovarian tissue, Tumor-P represents ovarian cancer in situ, and Tumor-O represents metastatic ovarian cancer of the omentum. **G** From left to right, results of the Pearson’s correlation analysis of immunohistochemical scores between ACSL1 and FSP1, 4-HNE and PTGS2, *P < 0.05 and ***P < 0.001 indicate statistical significance.

To address the role of ACSL1 in controlling protein degradation of FSP1, we found that ACSL1 participated in protein N-myristoylation by increasing myristic acid levels in cancer cells [14]. FSP1 is a potential candidate for myristoylation, as it contains a N-terminal glycine. Therefore, we speculated that myristoylation of FSP1 could be enhanced by upregulation of ACSL1 during spheroid formation. To determine the role of ACSL1 in the myristoylation of FSP1, we established ACSL1 overexpressing cells (ACSL1-WT) and ACSL1 mutated (ACSL1-MT) HM and NM cells. ACSL1 overexpression did not affect the level of N-myristoyltransferase 1 (NMT1), which suggests that the increase in FSP1 myristoylation was not due to changes in the level of NMT1 (Fig. 4A). FSP1 myristoylation was analyzed by western blotting with Click-iT chemistry pull-down myristoylated protein extracts. The results suggested that overexpression of ACSL1 (ACSL1-WT) can significantly increase myristoylation of FSP1 in NM cells compared to the vector control. Although mutation of ACSL1 (ACSL1-MT) could dramatically reduce myristoylation of FSP1 in both HM and NM cells (Fig. 4B). Previous studies have found that myristoylation of FSP1 promotes membrane localization and improves lipid peroxidation [8, 24]. To verify the distribution of FSP1, we detected the localization of FSP1 and E-cadherin by immunofluorescence staining, and the results showed that the colocalization of FSP1 and E-cadherin increased in the cytomembrane after the expression of ACSL1-WT compared to the vector control and the ACSL1-MT group (Fig. 4C). To further elucidate whether ACSL1 regulates the sensitivity of ferroptosis, the ferroptosis inducer Erastin and RSL3 were used. At high concentration of Erastin or RSL3, ferroptosis was significantly induced and cell proliferation was suppressed in all tested cell lines, even the effect on HM cells was minimal. But this effect were reversed in ACSL1-WT cells, but not in ACSL1-MT cells (Fig. 4D). Western blotting analysis also confirmed that ACSL1-WT overexpression increased the level of FSP1 protein after erastin treatment compared to the vector control and ACSL1-MT groups (Fig. 4E).

To explore the clinical significance of ACSL1 and FSP1, and of the ROS biomarkers, 4-HNE and PTGS2, in cancer metastases, tumors samples from patients with ovarian cancer were also tested by immunohistochemistry. 4-HNE, a specific lipid peroxidation product, has been reported to be the most sensitive marker of lipid peroxidation products [25]. PTGS2 cyclooxygenase can oxidize lysophospholipids, affecting phospholipid peroxidation, which is a biomarker of cellular ferroptosis [25]. ACSL1 and FSP1 expression was significantly higher in metastatic tumors in the omentum than in tumors of the normal ovary and in primary ovarian cancer, while the expression of 4-HNE and PTGS2 was lower in the omentum metastasis tumor (Fig. 4F). Pearson’s correlation analysis was performed and the results showed that the level of ACSL1 protein was positively correlated with the level of FSP1 protein, while ACSL1 was negatively correlated with the level of the 4-HNE and PTGS2 protein (Fig. 4G). The results indicated that the high expression of ACSL1 contributed to the metastasis of ovarian cancer and resisted the ferroptosis of cancer cells during the metastasis.

### Up-regulation of ACSL1 improved carboplatin resistance and metastasis by suppressing ferroptosis

**Fig. 5:**
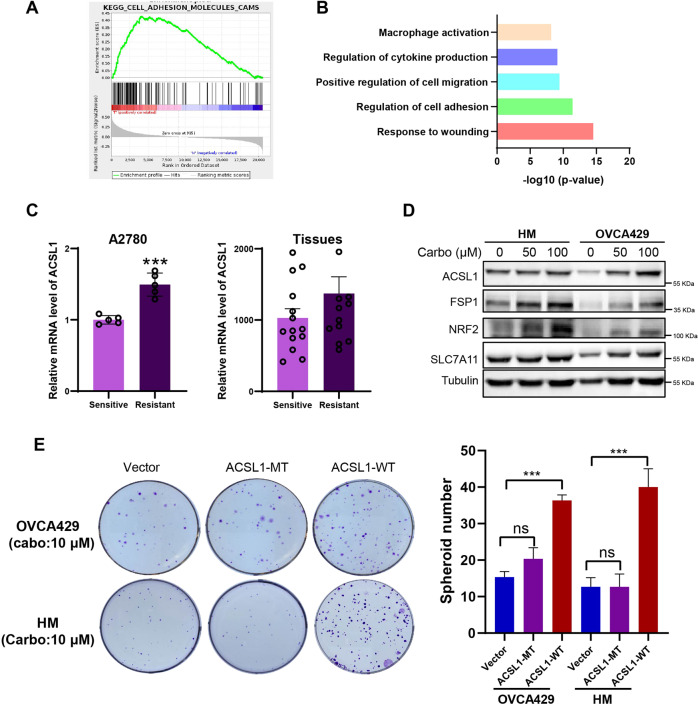
ACSL1 participates in carboplatin therapy for ovarian cancer by regulating cell ferroptosis. **A** GSEA analysis of the activation and expression of gene pathways in cancer tissues from patients with high and low ACSL1 expression. **B** GO analysis suggests that genes co-expressed with ACSL1 are involved in biological processes such as cell migration, adhesion, and secretion of immune factors. **C** Expression of ACSL1 gene mRNA in carboplatin-sensitive and carboplatin-resistant cancer tissues (right) and in the A2780 cell line (left). **D** After carboplatin treatment of HM and OVCA429 cells, the expression of ACSL1 and ferroptosis hallmark proteins was detected. **E** The spheroid formation ability of OVCA429 and HM cell after expression ACSL1-WT or ACSL1-MT vectors and treatment with carboplatin. The live spheroids were reseeded into standard 6-well plates and allowed to grow for a period of 7–10 days. Subsequently, they were stained with crystal violet for further analysis. ***P < 0.001 indicates statistical significance.

Platinum chemotherapy is the first-line treatment for ovarian cancer, but most patients develop resistance [5]. Cancer cells with significant changes in cell-cell interaction enhance spheroid formation as an important pathway to achieve chemotherapy resistance [26]. Using data from on the online Cancer Gene and Pathway Explorer database (https://cgpe.soic.iupui.edu/), GSEA analysis showed that tumor tissue from patients with ovarian cancer with high expression of ACSL1 was associated with significantly upregulated cell adhesion functions (Fig. 5A). Gene Ontology (GO) analysis also suggested cell adhesion genes were significantly upregulated and enriched in high ACSL1 tissues (Fig. 5B). Furthermore, the GEO data set also revealed that ACSL1 expression was increased in A2780 platinum-resistant cells (1.49-fold, *P* < 0.001) and was increased in carboplatin-resistant patients, although in the carboplatin-resistant cases the difference was not statistically significant (1.37 times, *P* = 0.201, Fig. 5C). Therefore, we proposed that the increase in ACSL1 and antioxidation during spheroid formation may be involved in the development of drug resistant cells in ovarian cancer cells. To verify this hypothesis, HM and OVCA429 cells were treated with carboplatin, leading to up-regulation of the protein level of ACSL1 and FSP1 (Fig. 5D) The expression of antioxidant-related proteins NRF2 and SLC7A11 also increased (Fig. 5D). Overexpression of ACSL1-WT rather than ACSL1-MT can reduce carboplatin cytotoxic and increase spheroid formation in ovarian cancer OVCA429 and HM cell lines (Fig. 5E). These data confirmed that carboplatin treatment could lead to activation of ACSL1 and the anti-ferroptosis pathway to enhance ovarian cancer carboplatin resistance.

## Discussion

Ovarian cancer is a highly aggressive malignant gynecological tumor with a high mortality rate. Since ovarian cancer spreads through the abdominal cavity, elucidating the mechanism of ovarian cancer survival in the abdominal cavity is an important mechanism to be addressed. Platinum-based drugs are widely used for the chemotherapy of ovarian cancer [27]. Platinum chemotherapy-sensitive patients can achieve a good response as a first-line drug. However, the probability of developing platinum resistance will increase significantly after multiple treatments [28].

Redox homeostasis is crucial for cell survival and the development of drug-resistant cancer cells. Studies have shown that when detached cancer cells are treated with platinum chemotherapy, they experience a stress state and surge of ROS formation [29]. However, the strong redox regulation ability of cancer cells ensures that cancer cells can withstand harsh conditions to survive [30]. Ferroptosis is a form of RCD caused by uncontrolled redox homeostasis. Compared to apoptosis, ferroptosis is considered a “metabolic cell death” pathway [31]. Therefore, metabolic changes in cancer cells are closely related to ferroptosis and targeting the ferroptosis pathway has been proposed for the development of next-generation chemotherapeutic drugs [6]. However, the mechanisms underlying the initiation and termination of ferroptosis, especially how cancer cells escape from ferroptosis, are unclear. Therefore, this study examined the correlation between metabolic reprogramming and the mechanism of ferroptosis and platinum resistance.

ACSL1 has been shown to participate in fatty acid activation and downstream catabolism or synthesis. Our previous report suggested that the increase in ACSL1 is associated with increased myristic acid production and enhance protein myristoylation. This report suggests that the increase in ACSL1 during spheroid formation could also enhance the canonical signaling axis of anti-peroxidation NRF2/SLC7A11/GPX4 by myristoylation of FSP1. Therefore, the absence of lipid peroxidation is beneficial for spheroid formation and maintains the intracellular redox homeostasis for cancer cells to escape ferroptosis. This explained why the sensitivity of HM cells with Erastin treatment was associated with the expression of ACSL1 (Fig. 2). This study also suggested that the expression of ACSL1 was positively correlated with the expression of FSP1 and inhibition of ferroptosis. Another study has reported that ACSL1 induces ferroptosis by mediating the integration of exogenous conjugated linolenic acid into phospholipids [13]. However, the regulation of endogenous lipid metabolism during ferroptosis is still unclear and more studies are required.

To date, there are four main pathways for cells to resist ferroptosis, three of which are focused on the plasma membrane (SLC7A11/GPX4, FSP1/CoQ10, and GCH1/BH4), while DHODH/CoQ10 works on the scavenging of mitochondrial lipid peroxidation [15]. By manipulating ACSL1 expression in HM cells and OVCA429 cells, we found that the expression of GPX4 and SLC7A11 in the two cell lines was not consistent, suggesting that ACSL1 may indirectly regulate the antioxidant signaling pathway SLC7A11/GPX4. These data are consistent with a report suggesting that FSP1 is an anti-lipid peroxidation protein independent of GPX4, and its activation depends on myristoylation modification and subsequent cell membrane localization [8, 24]. FSP1 prevents lipid oxidation by reducing CoQ10, thus inhibiting ferroptosis, and that the pro-apoptotic and cytostatic effect of FSP1 on ferroptosis depends on cellular location. FSP1 localized in the nucleus promotes cell apoptosis, whereas FSP1 is localized in the mitochondria when cells are under oxidative stress. The recruitment of FSP1 to the cell membrane allows it to utilize NADPH to reduce lipid peroxidation [7]. Our experimental results confirmed that ACSL1 increases the myristoylation of FSP1 and enhanced its recruitment to the membrane. In addition, changes in FSP1 cellular membrane localization could significantly enhance the stability of FSP1 by preventing FSP1 protein degradation and therefore could inhibit ovarian cancer ferroptosis.

**Fig. 6:**
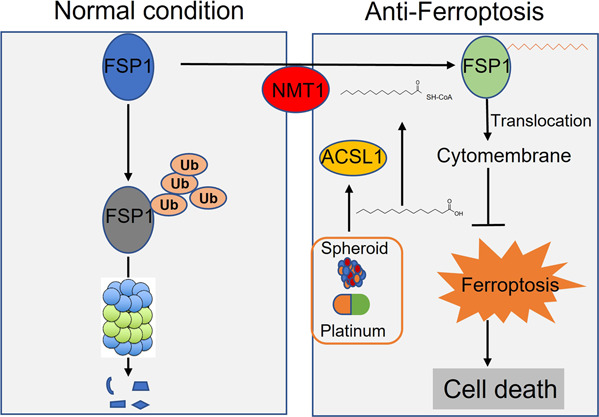
ACSL1 involvement in anti-ferroptosis in ovarian cancer. During the process of metastasis in the peritoneal cavity, ovarian cancer cells form multicellular spheroids that can withstand both nutrient deprivation and platinum-based chemotherapy-induced production of reactive oxygen species (ROS). To overcome the high levels of ROS-induced cell ferroptosis, cancer cells upregulate ACSL1, which promotes the translocation of ferroptosis suppressor protein-1 (FSP1) to the cell membrane by increasing FSP1 myristylation.

Platinum chemotherapy induces cancer cell apoptosis and ferroptosis by inducing massive ROS release and then damage to cellular DNA or to the cell membrane [32, 33]. Recalcitrant cancer cells can enforce redox homeostasis through metabolic reprogramming, resulting in acquired drug resistance of tumor cells [1]. However, the role of lipid metabolism molecules in tumor resistance has rarely been studied, and the relationship between platinum resistance and lipid metabolism is also unclear. This report demonstrated that activation of the ACSL1/FSP1 cellular anti-ferroptosis pathway mentioned above after carboplatin treatment reduces the sensitivity of cancer cells to platinum chemotherapy and promotes cell survival (Fig. 6). but the specific molecular mechanism involved in platinum resistance in ovarian cancer requires further study. Therefore, our work revealed a potential therapeutic target for ovarian cancer and a theoretical basis for clinical transformation to overcome platinum resistance in ovarian cancer.

## Methods and Materials

### Antibodies and reagents

The Cell Counting Kit 8 (CCK8, cat:C0038) and puromycin (cat:ST551) were purchased from Beyotime, China; Reactive Oxygen Fluorescent Probe (DCFHDA, cat: BL714A) was obtained from Biosharp, China; RSL3 (1S,3R-RSL3, cat:A15865) was from Adooq Bioscience, USA. Cycloheximide (CHX, cat:HY-12320), 3-methylamine (3-MA,cat:HY-19312), deferoxamine mesylate (DFO, cat:HY-B0988) were purchased from MCE, China; Necrostatin-1(cat:A4213), ferrostatin-1(cat:A4371), erastin(cat:B1524), MG-132(cat:A2585), ZVAD-FMK(cat:A1902) were purchased from APEXBIO, USA; anti-NRF2 mAb, anti-GPX4 mAb (cat:59735) were purchased from CST, USA; anti-AIFM2 polyclonal antibody(cat:abs138156) was from Absin, China; anti-SLC7A11 polyclonal antibody(cat:26864-1-AP), anti-PTGS2 polyclonal antibody(cat: 66351-1-Ig), anti-alpha tubulin antibody(cat:11224-1-AP), and anti-β-actin antibody(cat: 81115-1-RR) were purchased from PTG, China; anti-4-HNE antibody(cat:MAB3249) was purchased from R&D Systems, USA; Protein A/G Mix magnetic beads kit (cat: 88802) was from Thermo fisher, USA; Myristic acid alkyne(cat: 82909-47-5), Biotinamine (Azide-PEG3-biotin conjugate, cat:762024) were purchased from Santa Cruz, USA; The Click-it Chemistry Kit(cat: C10276), C11 BODIPY581/591(cat:D3861), Streptavidin (DynabeadsTM, cat:65001), and anti-TfR1 polyclonal antibody (cat:14-0719-82), anti-SREBP1 (cat:MA5-11685) were purchased from Thermo Fisher, USA.

### Cell culture

Human epithelial ovarian cancer cell lines, including OVCA429, were acquired from Prof. George Tsao (The University of Hong Kong). The isogenic metastatic cell line model and highly metastatic (HM) and non-metastatic (NM) cell lines were kindly gifted by Professor Alice Wong (The University of Hong Kong). All cell lines were maintained with DMEM with 10% FBS culture medium with penicillin/streptomycin in a humidified incubator with 5% CO_2_. These cell lines have been authenticated using STR profiling and tested for mycoplasma contamination, with results indicating cell are correct with no mycoplasma contamination.

### Patient samples

This study protocol was approved by the Affiliated Research Ethics Committee of Guangdong Medical University (YJYS2021105). A total of 72 clinical tissue samples collected from the Department of Obstetrics and Gynecology, Affiliated Hospital of Guangdong Medical University from March 2020 to December 2021 were selected, including 23 normal ovarian tissues, 17 primary ovarian cancer tissues, and 32 metastatic ovarian cancer tissues. Informed consent and consent for publication were obtained from all subjects before their participation in the study. Tissue samples harvested from surgery were washed with PBS, then snap-frozen in liquid nitrogen and stored at −80 °C. Inclusion criteria for the experimental group were the following: (1) patients diagnosed with ovarian cancer for the first time, with no other treatment history, and no other disease or tumor; (2) patients who did not receive hormone therapy, radiotherapy, or chemotherapy before surgery. Exclusion criteria of the experimental group consisted of (1) patients whose tumors originated outside the ovary and (2) patients whose medical records were incomplete.

### Plasmid and lentivirus packaging

The effective interference sequence 315# (5’-GTGGGTGATTATTGAACAA-3’), described in a previous study was inserted into the lentiviral vector pLK0.1 and was used to silence ACSL-1 (ACSL1-Knockdown, ACSL1-KD) [14]. The pCDH-CMV-MCS-EF1α-Puro lentiviral vector pCDH-CMV-MCS-EF1-Puro was used for ACSL1 cDNA gene overexpression (ACSL1-Wild type, ACSL1-WT). Based on the ACSL1-WT sequence, the active enzyme site of ACSL1 was mutated at positions S277A and K675A by site-directed mutagenesis to obtain the ACSL1 mutant (ACSL1-mutant, ACSL1-MT). All lentiviral vectors were packaged with DVPR and VSVG packaging plasmids, and polyethylenimine (PEI) was used for cell transfection. The ratio of target plasmid: VSVG: DVPR was 1:0.3:1 and was transfected into HEK293T cells in six-well plates with a total amount of DNA equal to 2.5 μg/well. The packaged virus was obtained at 24 and 48 h and cell debris was removed using a 0.45 μm filter. The packaged virus and the fresh culture medium were mixed 1:1 for ovarian cancer cell infection. After 24 h of cell infection, cells were tested with 1 μg/mL puromycin for 3–5 days, and western blotting was used to verify protein expression after overexpressing or knocking down ACSL1 expression.

### Cell Counting Kit-8 assay

Target cells were seeded in a 96-well plate at a density of 3000 cells/well. A 10 μL volume of CCK8 solution was added to each well and the cells were incubated for 1 h. The absorbance of the substate was measured in a microplate reader at OD450. Cell viability was recorded using the following formula: Relative viability = 100% (Target group OD450 – Blank well OD450) / (Control group OD450 – Blank well OD450).

### 2,7-Dichlorofluoroscin diacetate assay

Cells in monolayer or in spheroid formation were trypsinized and washed three times with PBS to remove cell debris. Cells were counted and 1 × 10^5^ cells were subjected to 2,7-dichlorofluoroscin diacetate (DCFHDA) labeling. The DCFHDA probe was used at a 1:1000 dilution in staining buffer and cells were incubated within the staining buffer at room temperature for 30 min to label the cells. After three washes with PBS to remove the unlabeled probe, the cells were resuspended with PBS and the level of ROS was detected in the green channel (488 nm/535 nm excitation/emission) by flow cytometry (Attune NxT, Invitrogen, USA).

### Click-It chemistry

Myristic acid alkyne was added to the corresponding cells and treated for 6–12 h to allow myristoylated protein labeling. The cells were collected and lysed with RIPA buffer, centrifuged to remove the supernatant, and biotinylated alkyne was added. The cells were then treated according to the Click-It chemistry reaction kit instructions (C10269, Thermo Fisher), which enables the rapid formation of crosslinks between azides and alkynes. After centrifuging and washing, the labelled proteins were pulled down by avidin magnetic beads, and the amount of the target protein myristoylation was detected by western blotting.

### Confocal imaging

Stable OVCA429 cell lines were inoculated on sterilized coverslips in a 12-well cell culture plate. After fixation with 4% paraformaldehyde (5 min), the cells were washed three times with PBS buffer (5 min each time) and permeabilized with 0.5% TritonX-100 for 10 min. 1% BSA was added to block the antigen for 30 min. The primary antibody FSP1 (abs138156, Absin, China; 1:200) and E-cadherin (abs158305, Absin, China; 1:200) were incubated with the samples overnight at 4 °C in a humidified chamber. After incubation, the coverslips were washed with PBS and incubated with Alexa Fluor 594 (abs20021, Absin, China; 1:100) and Alexa Fluor 488 (abs20014, Absin; 1:100) fluorescently labeled secondary antibodies at room temperature in a dark box for 1 h. The nuclei were stained with DAPI before covering the coverslips with anti-fluorescence quenching mounting medium (P0126, Beyotime, China). The slides were observed and recorded using a confocal fluorescence microscope (Olympus, FV3000, Japan).

### Co-immunoprecipitation

HM cells were harvested in the logarithmic growth phase and inoculated at 2 × 10^6^ cells/well in a 10-cm plate. When approximately 90% confluence was reached, the protein was extracted (all procedures were performed on ice) by RIPA buffer supplemented with phenylmethylsulfonyl fluoride (PMSF). Cells were collected with a scraper and incubated on ice for 30 min. After centrifugation at 12,000 x g at 4 °C for 10 min, the supernatant was obtained as the extracted protein. Protein concentration was quantified using a BCA protein quantification kit. A total of 20 µg of extracted protein solution was denatured and stored as an input control and the remaining samples were used for immunoprecipitation. A 30 µL volume of magnetic beads was used for 2 mg of protein precipitation. Nonspecific binding was removed by 2 µg IgG and incubated for 4–6 h at 4 °C with 30 µL of magnetic beads (88802, Thermo Fisher, USA). The samples were transferred to a new Eppendorf tube with magnetic beads. The corresponding IP antibody (ACSL1 or FSP1) was added and incubated at 4 °C overnight. IP with IgG was used as a control. The prepared samples from each group were placed in a rotary mixer for rotation and incubated. IP products were washed with PBST and finally resuspended in 40 µL of 2× loading buffer for the western blotting.

### Ubiquitination of FSP1 analysis

HA-Ub and Myc-FSP1 expression plasmids together with Flag-ACSL1-WT or Flag-ACSL1-MT were co-transfected into HEK293T cells. Cells were treated with 20 μM MG132 44 h post-transfection for 4 h and then lysed using lysis buffer (P0013, Beyotime). The cell lysates were immunoprecipitated by anti-Myc magnetic beads (P2118, Beyotime) overnight at 4°C. The immunocomplexes were subjected to western blotting. The ubiquitin level of FSP1 was detected using an anti-HA antibody (cat:3724, CST). The expression of FSP1 and ACSL1 was detected by anti-FSP1 and anti-ACSL1 antibodies, respectively.

### Immunohistochemistry

Ovarian tumor samples were fixed in 4% paraformaldehyde. The tissue was dehydrated, embedded in paraffin, and sectioned for immunohistochemistry. The stained samples were observed and photographed under a microscope. A positive reaction was scored by the appearance of brownish yellow or tan particles, and the intensity of the staining was scored. Positive scoring was divided into weak positive, medium positive, and strong positive. A negative result was scored as “0 points”, weak positive was light yellow and scored as “+ or 1 point”, medium positive was brown and scored “++ or 2 points”, and strong positive was tan and scored “+++ or 3 points”. The average score of three independent investigators was used to define the target protein level.

### Non-targeted metabolomic analysis

Non-targeted lipidomic analysis was performed as described previously [15]. Briefly, cells were thawed on ice and metabolites were extracted using lipid extraction buffer (isopropanol: acetonitrile: water = 2:1:1). A 100 mg sample was extracted with 1 mL of precooled buffer, incubated for 10 min, and stored overnight at −20 °C. Supernatants were then transferred to new 96-well plates, and pooled QC samples were prepared by combining 10 μL of each extraction mixture. Chromatographic separation was performed using an Acquity UPLC System, with a Kinetex UPLC C18 column and a flow rate of 0.3 mL/min. Metabolites were detected using a high-resolution TripleTOF6600 mass spectrometer in positive and negative ion modes. Data pretreatment and identification of ions were performed using XCMS, CAMERA, and metaX R software tools.

### Bioinformatics analysis

Keywords ‘ovarian cancer’ and ‘platinum’ were used to search the Gene expression omnibus (GEO). We collected the two datasets GSE15709 and GSE51373 containing the gene expression profiles of cell lines and patient tissues. We extracted the expression of the ACSL1 gene from the transcriptional expression matrix. Gene set enrichment analysis (GSEA) and Gene ontology (GO) analyses were performed using an online bioinformatics tool (https://cgpe.soic.iupui.edu).

### Statistical analysis

Prism software (version 8.0, GraphPad) was used to perform statistical analysis. Data are presented as mean ± standard error. A two-group comparison was conducted and analyzed using Student’s t-tests. We used ANOVA (Analysis of Variance) to compare the means of multiple groups and post-hoc test (Bonferroni) employed. The equal variances were checked prior to analysis to ensure the validity of the results. All t tests were two-sided and *P* < 0.05 were considered significant.

## Supplementary information

Original Data File

## Data Availability

All data generated in this study are available upon reasonable request. Please contact the corresponding author for any data inquiries.

## References

[1] Tan Y, Li J, Zhao G, Huang K-C, Cardenas H, Wang Y (2022). Metabolic reprogramming from glycolysis to fatty acid uptake and beta-oxidation in platinum-resistant cancer cells. Nat Commun.

[2] Lee M, Song BR, Kim DH, Ha J, Lee M, Choi SJ (2020). Up-Regulation of Superoxide Dismutase 2 in 3D Spheroid Formation Promotes Therapeutic Potency of Human Umbilical Cord Blood-Derived Mesenchymal Stem Cells. Antioxidants (Basel, Switzerland).

[3] Robinson M, Gilbert SF, Waters JA, Lujano-Olazaba O, Lara J, Alexander LJ (2021). Characterization of SOX2, OCT4 and NANOG in Ovarian Cancer Tumor-Initiating Cells. Cancers.

[4] Basuli D, Tesfay L, Deng Z, Paul B, Yamamoto Y, Ning G (2017). Iron addiction: A novel therapeutic target in ovarian cancer. Oncogene.

[5] Davis A, Tinker AV, Friedlander M (2014). “Platinum resistant” ovarian cancer: What is it, who to treat and how to measure benefit?. Gynecologic Oncol.

[6] Li J, Cao F, Yin H-L, Huang Z-J, Lin Z-T, Mao N (2020). Ferroptosis: Past, present and future. Cell Death Dis.

[7] Yan HF, Zou T, Tuo QZ, Xu S, Li H, Belaidi AA (2021). Ferroptosis: Mechanisms and links with diseases. Signal Transduct Target Ther.

[8] Doll S, Freitas FP, Shah R, Aldrovandi M, da Silva MC, Ingold I (2019). FSP1 is a glutathione-independent ferroptosis suppressor. Nature.

[9] Lei G, Zhuang L, Gan B (2022). Targeting ferroptosis as a vulnerability in cancer. Nat Rev Cancer.

[10] Tesfay L, Paul BT, Konstorum A, Deng Z, Cox AO, Lee J (2019). Stearoyl-CoA Desaturase 1 Protects Ovarian Cancer Cells from Ferroptotic Cell Death. Cancer Res.

[11] Ellis JM, Li LO, Wu PC, Koves TR, Ilkayeva O, Stevens RD (2010). Adipose acyl-CoA synthetase-1 directs fatty acids toward beta-oxidation and is required for cold thermogenesis. Cell Metab.

[12] Magtanong L, Ko PJ, To M, Cao JY, Forcina GC, Tarangelo A (2019). Exogenous Monounsaturated Fatty Acids Promote a Ferroptosis-Resistant Cell State. Cell Chem Biol.

[13] Beatty A, Singh T, Tyurina YY, Tyurin VA, Samovich S, Nicolas E (2021). Ferroptotic cell death triggered by conjugated linolenic acids is mediated by ACSL1. Nat Commun.

[14] Zhang Q, Zhou W, Yu S, Ju Y, To SKY, Wong AST (2021). Metabolic reprogramming of ovarian cancer involves ACSL1-mediated metastasis stimulation through upregulated protein myristoylation. Oncogene.

[15] Kuhl C, Tautenhahn R, Böttcher C, Larson TR, Neumann S (2012). CAMERA: An integrated strategy for compound spectra extraction and annotation of liquid chromatography/mass spectrometry data sets. Anal Chem.

[16] Cai Q, Yan L, Xu Y (2015). Anoikis resistance is a critical feature of highly aggressive ovarian cancer cells. Oncogene.

[17] Sawyer BT, Qamar L, Yamamoto TM, McMellen A, Watson ZL, Richer JK (2020). Targeting fatty acid oxidation to promote anoikis and inhibit ovarian cancer progression. Mol cancer Res: MCR.

[18] Frahm JL, Li LO, Grevengoed TJ, Coleman RA (2011). Phosphorylation and Acetylation of Acyl-CoA Synthetase- I. J Proteom Bioinforma.

[19] Li S, Lu Z, Sun R, Guo S, Gao F, Cao B (2022). The Role of SLC7A11 in Cancer: Friend or Foe?. Cancers.

[20] Rubinsztein DC (2006). The roles of intracellular protein-degradation pathways in neurodegeneration. Nature.

[21] Hou W, Xie Y, Song X, Sun X, Lotze MT, Zeh HJ (2016). Autophagy promotes ferroptosis by degradation of ferritin. Autophagy.

[22] Yang J, Stack MS (2020). Lipid regulatory proteins as potential therapeutic targets for ovarian cancer in obese women. Cancers.

[23] Wen Y-A, Xiong X, Zaytseva YY, Napier DL, Vallee E, Li AT (2018). Downregulation of SREBP inhibits tumor growth and initiation by altering cellular metabolism in colon cancer. Cell Death Dis.

[24] Bersuker K, Hendricks JM, Li Z, Magtanong L, Ford B, Tang PH (2019). The CoQ oxidoreductase FSP1 acts parallel to GPX4 to inhibit ferroptosis. Nature.

[25] Chen X, Comish PB, Tang D, Kang R (2021). Characteristics and Biomarkers of Ferroptosis. Front Cell Dev Biol.

[26] Liao J, Qian F, Tchabo N, Mhawech-Fauceglia P, Beck A, Qian Z (2014). Ovarian cancer spheroid cells with stem cell-like properties contribute to tumor generation, metastasis and chemotherapy resistance through hypoxia-resistant metabolism. PLOS ONE.

[27] Baert T, Ferrero A, Sehouli J, O’Donnell DM, González-Martín A, Joly F (2021). The systemic treatment of recurrent ovarian cancer revisited. Ann Oncol.

[28] van Zyl B, Tang D, Bowden NA (2018). Biomarkers of platinum resistance in ovarian cancer: What can we use to improve treatment. Endocr-Relat Cancer.

[29] Kleih M, Böpple K, Dong M, Gaißler A, Heine S, Olayioye MA (2019). Direct impact of cisplatin on mitochondria induces ROS production that dictates cell fate of ovarian cancer cells. Cell Death Dis.

[30] Ding DN, Xie LZ, Shen Y, Li J, Guo Y, Fu Y (2021). Insights into the role of oxidative stress in ovarian cancer. Oxid Med Cell Longev.

[31] Li S, Huang Y (2022). Ferroptosis: an iron-dependent cell death form linking metabolism, diseases, immune cell and targeted therapy. Clin Transl Oncol: Off Publ Federation Span Oncol Soc Natl Cancer Inst Mex.

[32] Marullo R, Werner E, Degtyareva N, Moore B, Altavilla G, Ramalingam SS (2013). Cisplatin induces a mitochondrial-ROS response that contributes to cytotoxicity depending on mitochondrial redox status and bioenergetic functions. PLoS One.

[33] Perillo B, Di Donato M, Pezone A, Di Zazzo E, Giovannelli P, Galasso G (2020). ROS in cancer therapy: The bright side of the moon. Exp Mol Med.

