# Supplementary figures and images for: ACSL1-induced ferroptosis and platinum resistance in ovarian cancer by increasing FSP1 N-myristylation and stability
Source: Cell Death Discov. 2023 Mar 8;9:83. doi: 10.1038/s41420-023-01385-2 (PMC9992462; doi:10.1038/s41420-023-01385-2)

Fig.1F

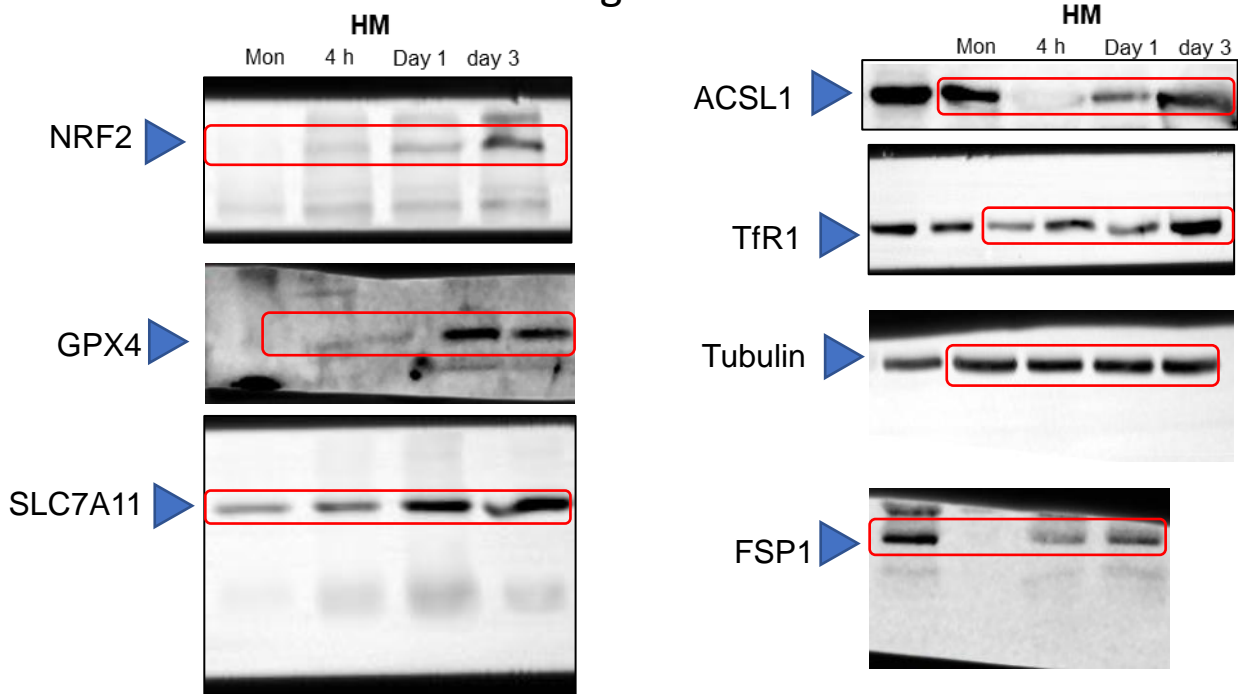

Fig.2A

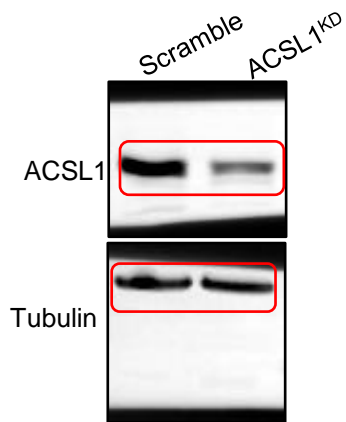

Fig.2B

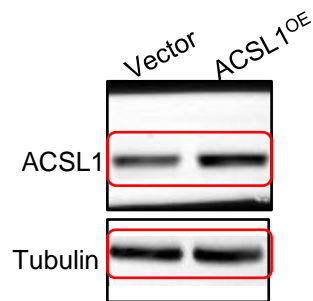

Fig.2F

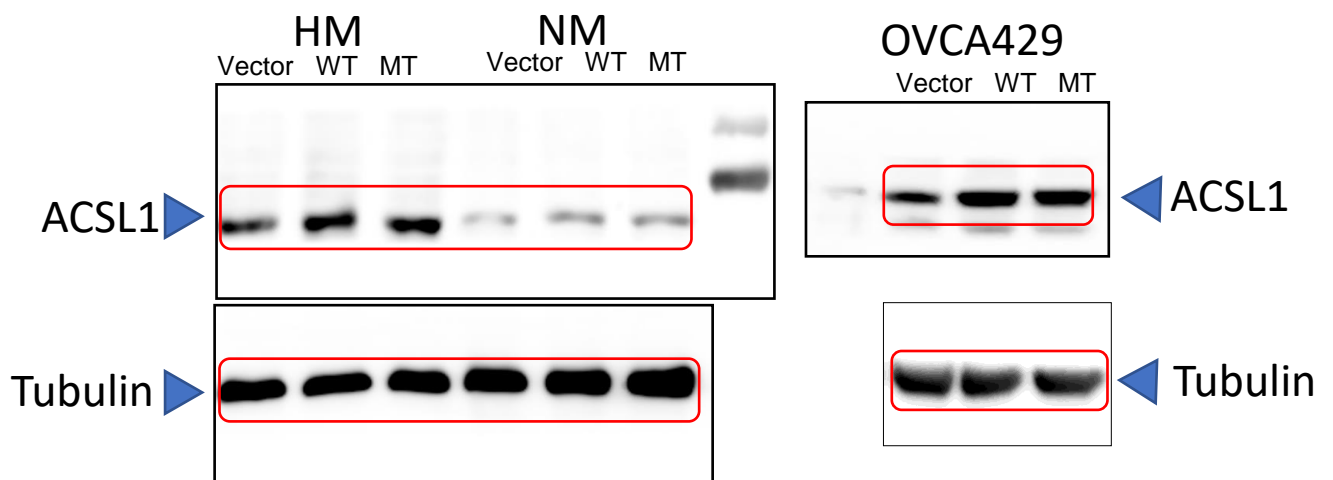

Fig.3A

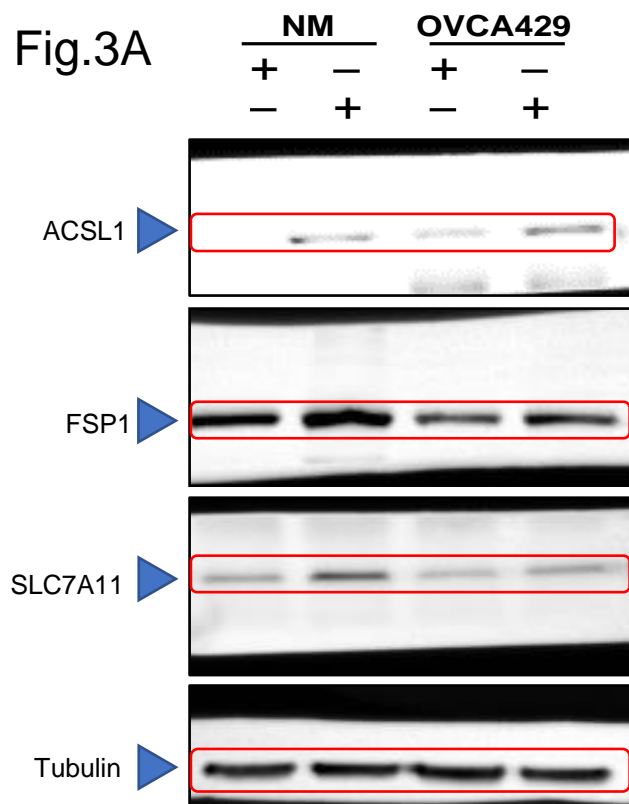

Fig.3B

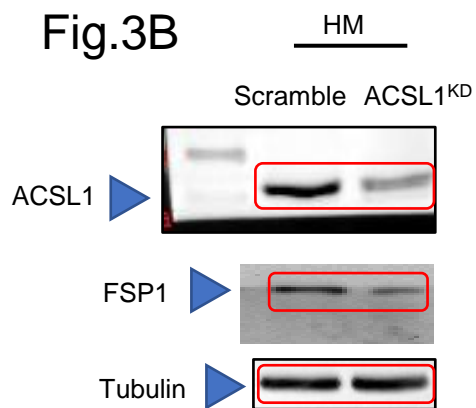

Fig.3C

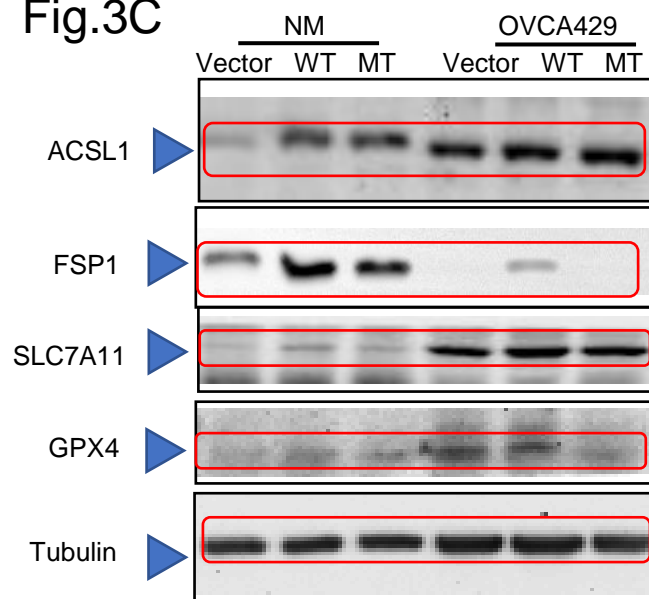

Fig.3D

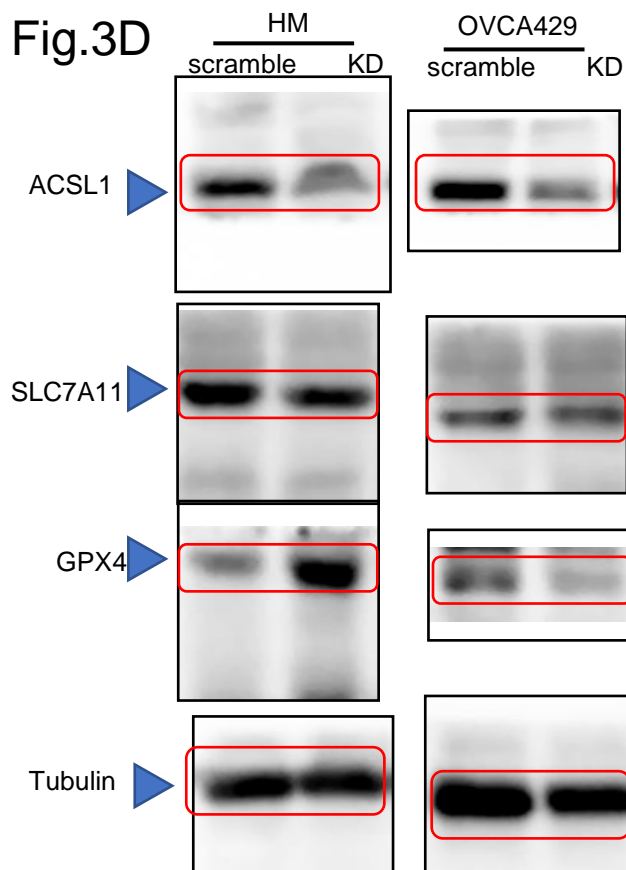

Fig.3E

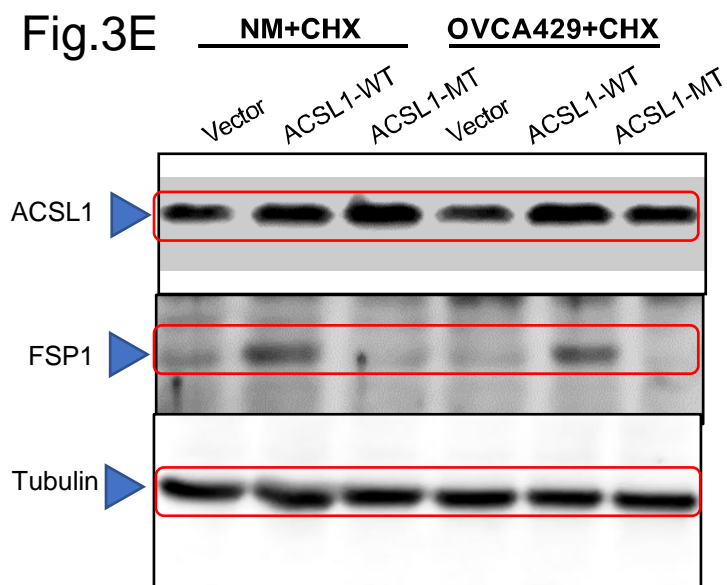

Fig.3F

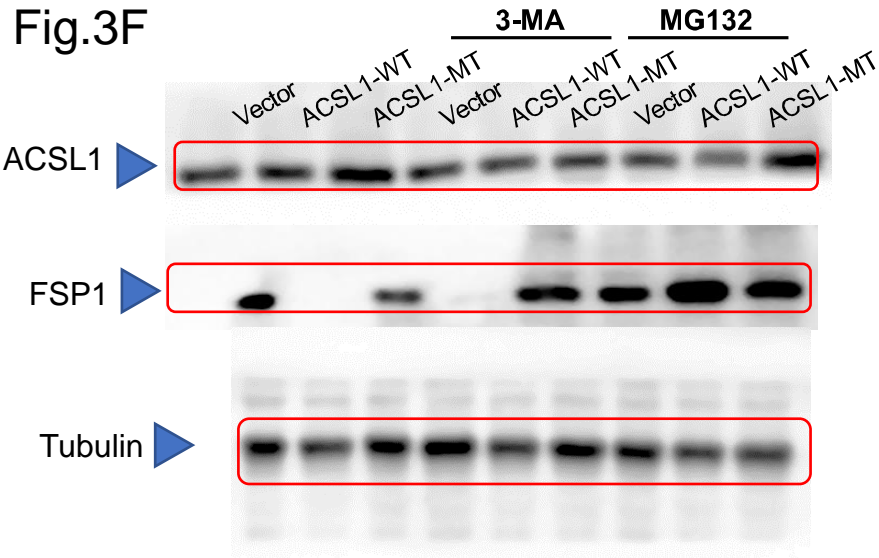

Fig.3G

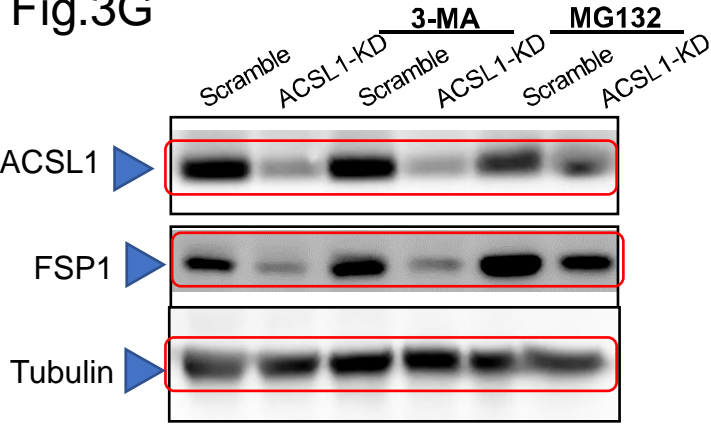

Fig.3H

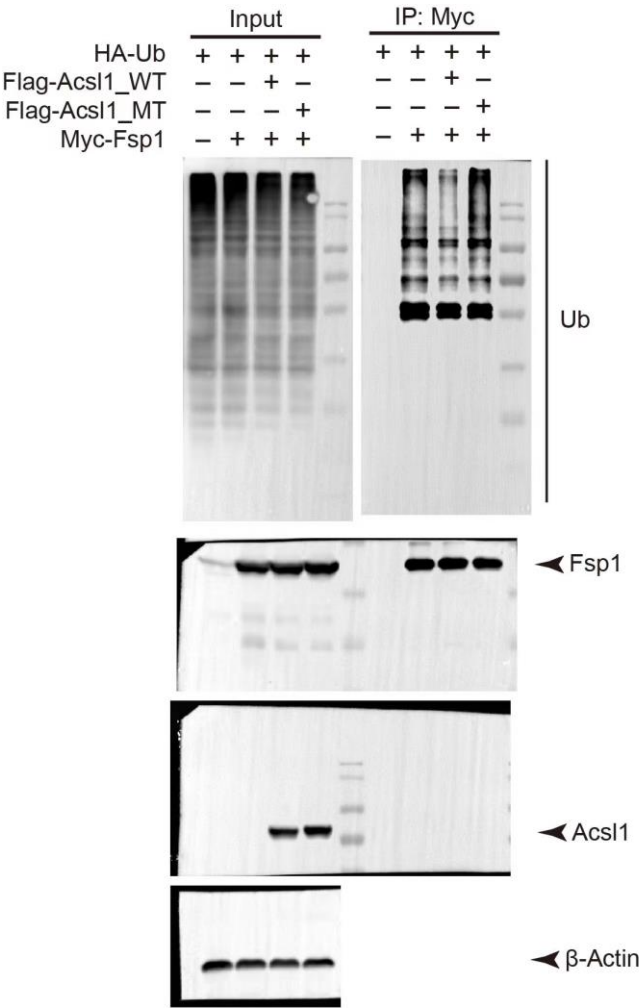

Fig.3J

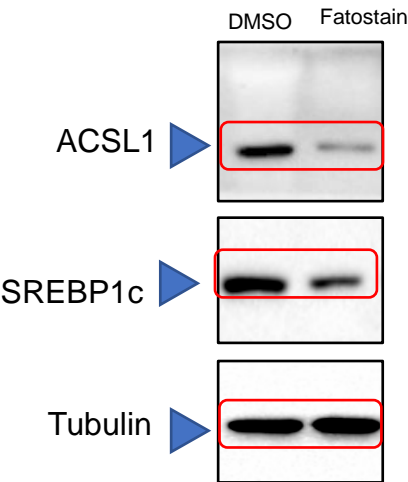

Fig.4A

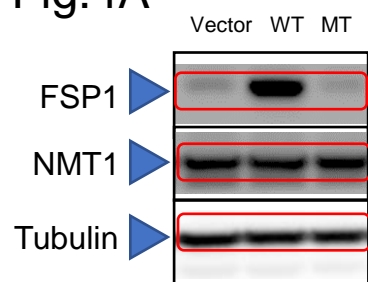

Fig.4B

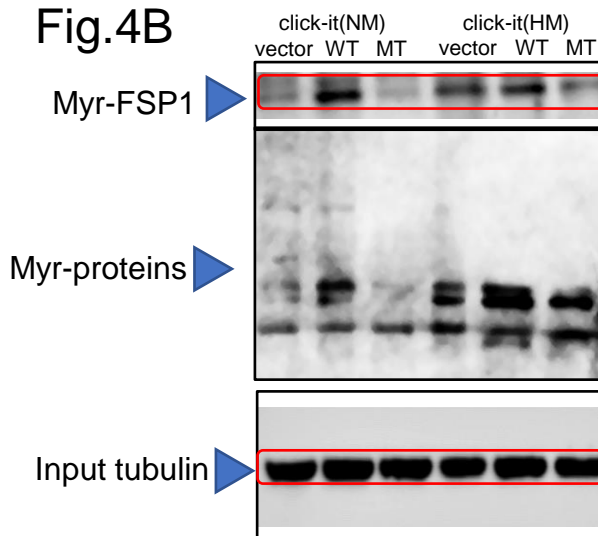

Fig.4C

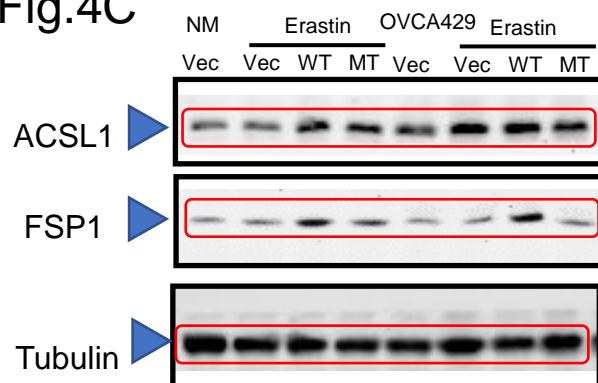

Fig.5D

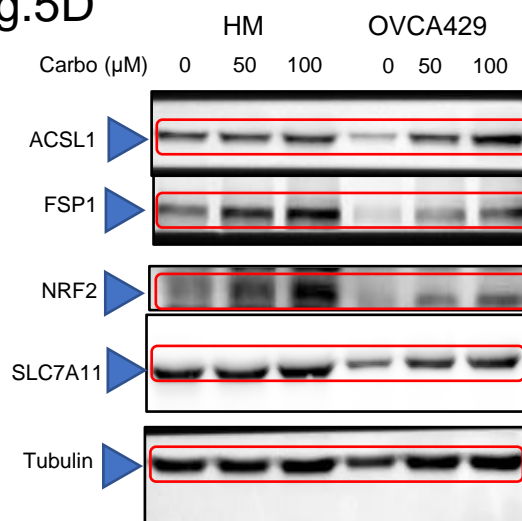

Supplement: Supplementary file 1 — Original Data File [file 41420_2023_1385_MOESM1_ESM.pdf]
